# *Streptococcus mutans* detection in saliva and colostrum samples

**DOI:** 10.31744/einstein_journal/2019AO4515

**Published:** 2019-01-28

**Authors:** Camilla Beatriz da Silva, Marcelly Milhomem Mendes, Bárbara Rocha Rodrigues, Thiago Lima Pereira, Denise Bertulucci Rocha Rodrigues, Virmondes Rodrigues, Virginia Paes Leme Ferriani, Vinicius Rangel Geraldo-Martins, Ruchele Dias Nogueira

**Affiliations:** 1 Universidade de Uberaba, Uberaba, MG, Brazil.; 2 Universidade Federal Triângulo Mineiro, Uberaba, MG, Brazil.; 3 Pós-Graduação em Saúde da Criança e do Adolescente, Faculdade de Medicina de Ribeirão Preto, Universidade de São Paulo, Ribeirão Preto, SP, Brazil.

**Keywords:** Streptococcus mutans, Saliva, Colostrum, Streptococcus mutans, Saliva, Colostro

## Abstract

**Objective:**

To detect *Streptococcus mutans* in colostrum and saliva of neonates and compare with its detection in saliva of mothers.

**Methods:**

Forty-three healthy women, full-term gestations with no complications, submitted to elective Cesarean section, and their newborns were included in the study. Samples were investigated by polymerase chain reaction to detect *S. mutans* in genetic material from the samples.

**Results:**

Approximately 16% of colostrum samples showed *S. mutans* , but not correlated with the presence of the bacteria in both samples of saliva. *S. mutans* was detected in 49 and 30% of saliva samples of mothers and neonates, respectively. There was a positive correlation in *S. mutans* detection between types of saliva. The number of maternal samples of saliva with detectable *S. mutans* was smaller in women receiving dental treatment during pregnancy. Tooth brushing, three times a day, influenced the detection of *S. mutans* in both the saliva and the colostrum.

**Conclusion:**

Although maternal saliva may present *S. mutans* , few samples of colostrum present the bacteria. The presence of bacteria in neonate saliva may be related to contact before birth. Dental treatment and hygiene habits seem to influence the detection of *S. mutans* in samples of maternal saliva and colostrum.

## RESUMO

**Objetivo:**

Detectar *Streptococcus mutans* no colostro e na saliva de recém-nascido, e comparar com a detecção na saliva da mãe.

**Métodos:**

Foram incluídos no estudo 43 mulheres saudáveis, com gestações a termo e sem complicações, que tiveram cesariana eletiva, e seus recém-nascidos. As amostras foram investigadas por reação de polimerase em cadeia para a detecção de *S. mutans* em material genético extraído das amostras.

**Resultados:**

Cerca de 16% das amostras de colostro apresentaram *S. mutans* , não sem correlação com a presença das bactérias em ambas amostras de saliva. *S. mutans* foi detectado em 49 e 30% das amostras de saliva das mães e recém-nascidos, respectivamente. Houve correlação positiva na detecção de *S. mutans* entre os tipos de saliva. O número de amostras de saliva materna com *S. mutans* detectável foi menor nas mulheres que receberam tratamento odontológico durante a gravidez. A escovação três vezes ao dia influenciou na detecção do *S. mutans* tanto no saliva quanto no colostro.

**Conclusão:**

Embora a saliva materna possa apresentar *S. mutans* , poucas amostras de colostro apresentam a bactéria. A presença de bactéria na saliva de neonatos pode estar relacionada ao contato antes do nascimento. O tratamento odontológico e os hábitos de higiene parecem influenciar na detecção de *S. mutans* em amostras de saliva e colostro maternos.

## INTRODUCTION

The genus *Streptococci* represents the majority of bacteria colonizing the oral cavity in the first months of life.^(^ ^1^ ^)^ The initial colonization of *Streptococcus mutans* occurs mainly after tooth eruption and is associated to the development of caries. *Streptococci mutans* colonize the dental surface, accumulate in the biofilm and produce acids that promote demineralization the tooth enamel.^(^ ^2^ ^)^ Dental caries is an infectious disease and a public health problem, especially in Brazil, because children are early colonized by *S. mutans* , even before tooth eruption.^(^ ^3^ ^)^ The early colonization can be associated with high consumption of sucrose, contact with highly infected individuals,^(^ ^4^ ^-^ ^6^ ^)^ and immaturity of mucosal immune system in children.^(^ ^7^ ^,^ ^8^ ^)^

The initial acquisition and establishment of the oral microorganisms occur by a microbial succession dependent on habits, contact with people and feeding. Breastfeeding is a common practice in Brazil, and it is necessary to investigate the colostrum components. It is known that colostrum has great bacterial diversity^(^ ^9^ ^)^ including *Streptococci* , *Staphylococci* , lactic acid bacteria and bifidobacteria,^(^ ^10^ ^)^ which can determine the body colonization of breastfed children. Intestinal microbiota of breastfed child is rapidly dominated by bifidobacteria^(^ ^11^ ^,^ ^12^ ^)^ and later, after dietary supplementation, is predominantly colonized by *Enterobacteriaceae* , *Streptococcus* , *Bacteroides* and *Clostridium* .^(^ ^11^ ^)^

Colostrum has several properties that benefit newborns, since it is an important source of nutritional and immunological components.^(^ ^13^ ^,^ ^14^ ^)^ The benefits of breast milk for numerous respiratory and intestinal infections have been amply described; however, for caries, they are unclear and controversial. There is evidence that dental caries is the negative outcome associated with breastfeeding, due to increased tooth decay in longer periods of breastfeeding.^(^ ^15^ ^,^ ^16^ ^)^ On the other hand, shorter duration of breastfeeding is suggested to be associated with increased risk for early childhood caries.^(^ ^17^ ^)^

Previous studies about the immunological analysis of colostrum from Brazilian women showed high levels of immunoglobulin A against *S. mutans* and its virulence antigens, which could contribute to the modulation of *S. mutans* infection.^(^ ^18^ ^,^ ^19^ ^)^ However, there is little information about the contribution of colostrum in the composition of oral colonization.

## OBJECTIVE

To evaluate the presence of *Streptococcus mutans* in colostrum samples and compare with saliva samples from mothers and newborns, associating the findings with data of oral health.

## METHODS

This study is an observational study with healthy neonates and mothers, who gave consent to take part. The Ethical Committee of the *Faculdade de Medicina de Ribeirão Preto* of *Universidade de São Paulo* approved this study under, CAAE: 02166713.4.0000.5145. Voluntary multiparous women were included in the study, submitted to clinical dental examination, and presented good oral (no caries or periodontal disease) and systemic health. They underwent elective Cesarean section in full-term gestation. Children with congenital malformations, perinatal hypoxia, intracranial hemorrhage, with length or weight incompatible with gestational age, or under antibiotic therapy were excluded from this study. Information on past medical/surgical and gestational history and oral hygiene was obtained through interviews with the mothers. Oral and maternal hygiene instructions were performed after the collections. All voluntaries (mothers and neonates) were referred for follow-up at the Dentistry Clinic of the University. Forty-three eligible samples came from a collection of 203 pregnant women who delivered at the maternity of *Hospital das Clinicas* of *Faculdade de Medicina de Ribeirão Preto* , who underwent oral cavity examination.

### Sample collection

On the first day of hospitalization, the pregnant women were interviewed, submitted to oral cavity examination, and non-stimulated saliva collection. Saliva of neonates was collected shortly after birth and prior to any contact with the mother, including the first breastfeeding, to avoid contamination with non-salivary components. Saliva samples were collected using sterile polypropylene transfer pipettes. A 250mM EDTA solution, pH 5.2 (Sigma, St Louis, MO, USA) was added to each sample prior to transport on ice to the laboratory, where they were stored at - 80ºC until analysis.

Samples of colostrum were collected by manual expression into sterile polypropylene Falcon tubes, on the first day of lactation. After collection, the samples were transported on ice to the laboratory, centrifuged at 1,300g, for 5 minutes, and stored at -80ºC until the laboratorial procedures.

### Detection of *Streptococcus mutans* in samples

DNA was extracted with the PowerLyzer PowerSoil DNA Isolation Kit (MO-BIO, Carlsbad, CA, USA) according to the manufacturer’s instructions. Firstly, the samples were incubated with 20mg/mL-^1^ lysozyme (Sigma, Tokyo, Japan) solution; containing 50mM Tris-HCl (pH 8.0) and 20mM EDTA, and incubated at 37^o^C, for 30 minutes. Samples were immediately transferred to a 1.5mL Eppendorf tube containing bead solution. After vortex mixing was carried out for 2 minutes to obtain bacterial suspension, which was transferred to a PowerLyzer Glass Bead Tube (MO-BIO, Carlsbad, CA, USA). The concentration of the purified PCR product was measured with a NanoDrop 2000 spectrophotometer (Thermo Scientific, Waltham, Massachusetts, USA). Sm479F/R primer pair (Sm479F: 5'-TCGCGAAAAAGATAAACAAACA-3'; and Sm479R: 5'-GCCCCTTCACAGTTGGTTAG-3')^(^ ^20^ ^)^ was purchased from Invitrogen (Tokyo, Japan). The StepOne™ Real-Time PCR System (Thermo Scientific, Waltham, Massachusetts, USA) performed Real-Time PCR. Each reaction tube contained reaction mixture, including 6.5µL SYBR Green Master Mix (Roche, Illinois, EUA), 1µL of each primer, 4.5µL of ultrapure water, and 2µL of DNA extracted from samples. A total of 45 cycles of 15 seconds were performed at 94°C for denaturation; 30 seconds at 56°C, for annealing; and 30 seconds at 72°C, for extension followed by a melting curve analysis of the PCR product. Two PCR tests were performed for all samples. *Streptococci mutans* (UA159) DNA was used as positive control.

### Statistical analysis

The differences in the frequencies of detection of *S. mutans* in the samples were obtained by the χ^2^ test. The correlation between positive detection of *S. mutans* and data of interview were tested by Pearson’s correlation coefficient. A p value <0.05 was considered statistically significant.

## RESULTS

*Streptococcus mutans* was not detected in most samples of colostrum (84%), and of saliva of neonates (70%) and of mothers (51%) ( Table 1 ). The comparative analysis between the frequencies of positive and negative detection of *S. mutans* in the samples showed that there were statistically significant differences between colostrum and mothers’ saliva, that is, maternal saliva presented more *S. mutans* than colostrum (χ^2^ test; p<0.05). There was a positive correlation between positive and negative detection of *S. mutans* between baby and maternal saliva (Spearman correlation; p<0.05; rs=0.36).

**Table 1:** Positive and negative samples of colostrum, maternal saliva, neonate saliva for detection of *Streptococcus mutans*

Samples n=43	Positive n (%)	Negative n (%)
Colostrum	7 (16)^*^	36 (84)^*^
Maternal saliva	21 (49)^*^	22 (51)^*^
Neonate saliva	13 (30)	30 (70)

^*^ χ^2^ test; p<0.003; q=10.37.

Most of the volunteers (70%) did not undergo dental treatment during pregnancy ( Table 2 ). Over 60% of women reported that regularly visited the dentist and brushed their teeth more than three times a day ( Table 2 ). Although mothers who did not undergo dental treatment during pregnancy had detectable *S. mutans* (69%), this difference was not statistically significant (p>0.05). The absence of dental treatment during gestation resulted in a higher number of positive samples in mothers’ saliva than in colostrum and baby saliva (p<0.05).

**Table 2:** Samples with positive and negative detection of *Streptococcus mutans* in colostrum, maternal saliva, and neonate saliva, according to oral health

Data	n (43)	Colostrum		Maternal saliva		Neonate saliva	
		
		Positive n (%)	Negative n (%)	Positive n (%)	Negativa n (%)	Positive n (%)	Negative n (%)
Oral health							
Visits to the dentist frequently	16	2 (13)	14 (87)	7 (44)	9 (56)	6 (38)	10 (62)
No	27	5 (19)*	22 (81)*	14 (52)*	13 (48)*	7 (26)	20 (74)
Dental treatment during gestation							
Yes	13	3 (23)	10 (77)	4 (31)	9 (69)	5 (38)	8 (62)
No	30	4 (13)^†^	26 (87)^†^	17 (57)^†‡^	13 (43)^†‡^	8 (27)^‡^	22 (73)^‡^
Number of tooth brushing per day							
1-2	16	4 (25)^§^	12 (75)^§^	10 (63)^§^	6 (37)^§^	6 (38)	10 (62)
Over to 3	27	3 (11)^¶^	24 (89)^¶^	11(41)^¶^	16 (59)^¶^	7 (26)	20 (74)

* Fisher’s exact test, p<0.05; ^†^ Fisher’s exact test, p=0.001; ^‡^ χ ^2^ test, p<0.001, q=18.47; ^§^ Fisher’s exact test, p<0.01; ^¶^ Fisher’s exact test, p<0.01.

There were no statistically significant differences in the frequency of *S. mutans* detection in the samples as per how many times mothers brush their teeth per day (p>0.05). There were also no statistically significant differences between colostrum and maternal and baby saliva (p>0.05). There was a statistically significant difference in the frequency of *S. mutans* detection when compared between colostrum and maternal saliva, for both tooth brushing once-to-twice a day, and three times a day (p<0.01).

## DISCUSSION

Analyze the presence of *S. mutans* by detection of genetic material in colostrum and saliva of mothers and newborns, as well as to compare the detection with data of oral health and hygiene habits. The Sm479F/R primer pair is highly specific and sensitive to identify *S. mutans* in either purified or mixed DNA samples.^(^ ^20^ ^)^ Although PCR was effective to detect *S. mutans* in samples and in the positive control, it must be considered that the genetic material detection using assays does not distinguish between living or non-living microorganisms, or bacterial fragments. All samples analyzed were from neonates born via Cesarean section, and the mode of delivery is a factor that influences the intestinal microbiota composition in early infancy.^(^ ^21^ ^)^

Although previous studies had detected species of *Streptococcus* abundantly in colostrum,^(^ ^22^ ^)^ in our study only 16% of the colostrum samples presented *S. mutans* . The entry pathways of bacteria through the mammary glands and exit via colostrum and mature milk are still unclear. There are some hypotheses about the presence of bacteria in the mammary glands, such as contact with the external environment, entry through other human body pathways, and a possible association of both hypotheses.^(^ ^23^ ^)^ External contamination can occur through contact of neonatal oral microbiota and skin microbiota during breastfeeding.^(^ ^24^ ^)^ This fact could explain the predominance of *Staphylococcus* and *Streptococcus* in breast milk, which are typical commensal of oral and skin microbiota.^(^ ^25^ ^,^ ^26^ ^)^ However, colostrum collection was performed before the first contact of the mother with the newborn, and after asepsis of breasts. Another pathway involves an internal bacterial route, which occurs when oral microorganisms reach the maternal intestine, leave by internalization in leukocytes (such as dendritic cells), migrate to the lymphatic vessels via blood circulation, and reach the mammary glands.^(^ ^27^ ^,^ ^28^ ^)^ Therefore, the minority of children received *S. mutans* via colostrum in the first day of life, but this process of bacterial translocation can be more intense, when the stimulus of suction and hormonal release stimulate better functioning of the mammary glands, and, consequently, secretion of bacteria that should be investigated.

The mouth is the site of the body with greater bacterial diversity as compared to vaginal or intestinal microbiota.^(^ ^29^ ^)^ As in other interfaces in our body, the oral mucosa is constantly fighting for tissue integrity. The immune system works to prevent the invasion of tissues by bacteria from the mouth.^(^ ^30^ ^)^ However, during gestation, there is an increased gingival permeability due to hormone changes, which can contribute to transfer of bacteria from the oral cavity to bloodstream. Some studies confirm presence of oral cavity bacteria in fetal tissues, especially those associated with periodontal disease, such as *Fusobacterium nucleatum* , in women who had preterm birth.^(^ ^31^ ^)^ Little is known about cariogenic bacteria from the maternal oral niche in immune development and fetal oral colonization.

The results shows that although the microorganism is present in some maternal saliva, it does not always enter the bloodstream, reaches the mammary glands, and is secreted in colostrum. Some mothers presented *S. mutans* in saliva but not in colostrum. And on contrary, the presence of *S. mutans* was observed in three samples of colostrum from mothers whose saliva had no *S. mutans* . Considering that *S. mutans* may be transient in the oral cavity, it is possible the bacteria were not present in the oral cavity upon collection. In general, 33% of infected women were able to transfer *S. mutans* through colostrum.

Of the newborns, 30% had *S. mutans* detectable in saliva, like their mothers. This suggests that exposure to bacteria and consequent colonization may occur in intrauterine life. *S. mutans* is transmitted to the fetus through the enteral-mammary via, reaching the oral cavity by swallowing the amniotic fluid. This mechanism could explain the immune stimulation and consequent production of specific salivary IgA against *S. mutans* in newborns, as found in previous studies.^(^ ^7^ ^,^ ^8^ ^)^ These findings differ from others that did not detect the bacteria at birth by the checkerboard technique,^(^ ^8^ ^)^ but only in children aged 5 to 11 months.^(^ ^3^ ^)^

The data obtained in the interviews with mothers provide important data regarding oral hygiene and behaviors, and their association with the presence of *S. mutans* in the samples. The mothers who stated not regularly visiting the dentist and not undergoing dental treatment during gestation presented higher detection of *S. mutans* in saliva. On the other hand, colostrum and baby saliva samples of these mothers had lower detection of *S. mutans* . The detection of *S. mutans* in patients brushing teeth more than three times a day was lower in all samples. The number of brushing times per day did not differ in positive or negative detection of *S. mutans* in each group of samples. The comparison between samples showed difference between colostrum and mother´s saliva. There were more colostrum samples with negative detection than saliva samples, when considering tooth brushing one or twice a day, and more than three times a day.

## CONCLUSION

*Streptococcus mutans* can be detected in a minority of colostrum samples. Behavioral data and hygiene habits appeared to influence the detection of *Streptococci mutans* in maternal saliva and colostrum samples. Dental treatment and caries prevention during pregnancy and after childbirth should be used to avoid the transmission of *Streptococci mutans* through maternal saliva and breast milk.
